# Image quality comparison of single-energy and dual-energy computed tomography for head and neck patients: a prospective randomized study

**DOI:** 10.1007/s00330-022-08689-4

**Published:** 2022-04-20

**Authors:** Andreas Bedernik, Wolfgang Wuest, Matthias Stefan May, Rafael Heiss, Michael Uder, Marco Wiesmueller

**Affiliations:** 1 Institute of Radiology, University Hospital Erlangen, Friedrich-Alexander-Universität Erlangen-Nürnberg (FAU), Maximiliansplatz 3, 91054 Erlangen, Germany; 2 Imaging Science Institute, University Hospital Erlangen, Friedrich-Alexander-Universität Erlangen-Nürnberg (FAU), Erlangen, Germany

**Keywords:** Dual-energy computed tomography, Automated tube voltage adaptation, Weighted average dual-energy images, Head and neck imaging

## Abstract

**Objectives:**

The aim of this study was to compare the quality of images obtained using single-energy computed tomography (SECT) performed with automated tube voltage adaptation (TVA) with dual-energy CT (DECT) weighted average images.

**Methods:**

Eighty patients were prospectively randomized to undergo either SECT with TVA (*n* = 40, ref. mAs 200) or radiation dose–matched DECT (*n* = 40, 80/Sn150 kV, ref. mAs tube A 91/tube B 61) on a dual-source CT scanner. Objective image quality was evaluated as dose-normalized contrast-to-noise ratio (CNRD) for the jugular veins relative to fatty tissue and muscle tissue and for muscle tissue relative to fatty issue. For subjective image quality, reproduction of anatomical structures, image artifacts, image noise, spatial resolution, and overall diagnostic acceptability were evaluated at sixteen anatomical substructures using Likert-type scales.

**Results:**

Effective radiation dose (ED) was comparable between SECT and DECT study groups (2.9 ± 0.6 mSv/3.1 ± 0.7 mSv, *p* = 0.5). All examinations were rated as excellent or good for clinical diagnosis. Compared to the CNRD in the SECT group, the CNRD in the DECT group was significantly higher for the jugular veins relative to fatty tissue (7.51/6.08, *p* < 0.001) and for muscle tissue relative to fatty tissue (4.18/2.90, *p* < 0.001). The CNRD for the jugular veins relative to muscle tissue (3.33/3.18, *p* = 0.51) was comparable between groups. Image artifacts were less pronounced and overall diagnostic acceptability was higher in the DECT group (all *p* = 0.01).

**Conclusions:**

DECT weighted average images deliver higher objective and subjective image quality than SECT performed with TVA in head and neck imaging.

**Key Points:**

*• Weighted average images derived from dual-energy CT deliver higher objective and subjective image quality than single-energy CT using automated tube voltage adaptation in head and neck imaging.*

*• If available, dual-energy CT acquisition may be preferred over automated low tube voltage adopted single-energy CT for both malignant and non-malignant conditions.*

## Introduction

Due to its wide availability, fast acquisition, and relatively low costs, computed tomography (CT) examinations are becoming more common worldwide [1, 2]. A major drawback of this technique is the use of x-rays, which are responsible for the majority of the radiation burden in diagnostic imaging and may increase individual cancer risk [3].

Different technical solutions like tube current modulation (TCM) and tube voltage adaptation (TVA) have been developed and introduced into clinical routine to minimize patient exposure while optimizing image quality [4], which is mainly defined by image noise and image contrast. With the widespread availability of powerful x-ray tubes, the combination of TCM and TVA is extensively used in clinical routine and generates images with high contrast-to-noise ratios (CNRs) [5]. Another promising technique to increase CNR is dual-energy CT (DECT). However, implementation of predicted low-kilo-electron-volt reconstructions (virtual monoenergetic images; VMI) in clinical routine is time consuming and therefore cost intensive. In contrast to VMI, weighted average image (WAI) reconstructions derived from DECT are instantly available at the scanner site. WAI are comparable to standard single-energy images acquired with 120 kV and offer a balanced trade-off between image contrast and image noise [6, 7]. In dual-source CT, the two x-ray tubes can be used either with the same tube voltage (single-energy CT; SECT) or with different tube voltages (DECT) [8]. While TCM is available in both settings, TVA is restricted to SECT. Thus, it remains unclear whether WAI reconstructions from DECT may generate equal or even superior image quality compared to single-energy images acquired with TVA on a dual-source CT scanner. This is of particular interest in head and neck imaging, which naturally suffers from low native contrast between soft tissues, which makes the administration of an iodine-based contrast agent almost indispensable [9].

The aim of this study was to evaluate the inter-individual image quality between dose-matched dual-energy-based WAI reconstructions and automatically selected low-tube-voltage single-energy CT images of the head and neck.

## Materials and methods

### Patient population

**Fig. 1:**
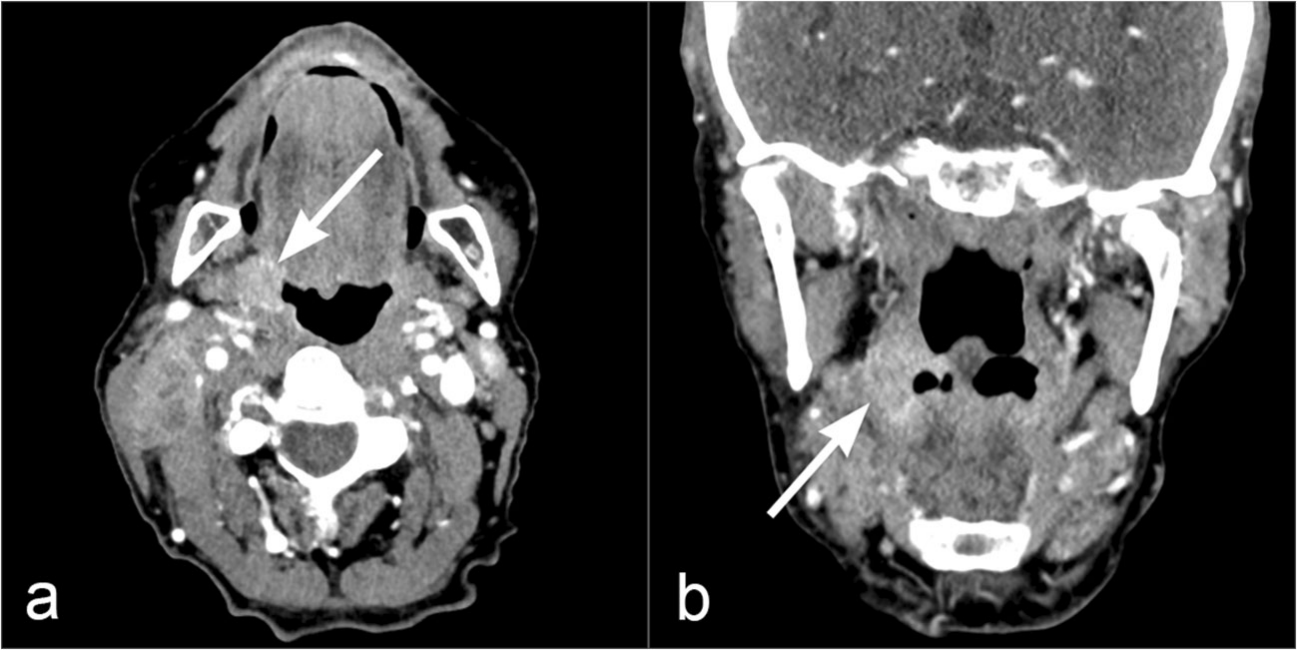
Example of image from the dual-energy CT (DECT) group. Images (**a**, **b**) are from a 59-year-old-male patient who underwent DECT. DECT clearly illustrates the oropharyngeal cancer on the right side (cT1N1M0, white arrows). Additionally, a lymph node metastasis was detected on the right side (no marking)

A total of 80 patients scheduled for a head and neck CT were included in this prospective, single-center study. The patients’ diseases covered a mixed spectrum including malignant and non-malignant conditions (Table 1); most patients with suspected malignancy underwent head and neck CT due to staging completion. Four patients had a primary malignancy in the head and neck region (Fig. 1). Patients were randomly assigned to either the DECT or the SECT study group prior to examination (40 patients per study group). Subjects with contraindications for CT imaging with intravenous administration of iodine contrast agents, such as history of allergies or intolerance to iodine contrast agents, nephropathy grade 4 or higher (estimated glomerular filtration rate < 30 mL/min/1.73 m^2^), hyperthyroidism, age < 18 years, and pregnancy, were excluded. All patients signed written informed consent for CT examination and study participation. The study was approved by the local Institutional Review Board and adheres to the Health Insurance Portability and Accountability Act (HIPAA) criteria and the Declaration of Helsinki.

**Table 1:** Demographic and clinical data of the patients studied. The patients are listed in the order in which they were examined in both SECT and DECT groups with consecutive numbering

Patient	Gender (F: female; M: male)	Age (years)	Suspected disease
SECT group			
1	F	61	Staging completion
2	F	79	Staging completion
3	F	76	Soft tissue inflammation
4	M	66	Staging completion
5	M	78	Staging completion
6	F	65	Staging completion
7	M	68	Soft tissue inflammation
8	M	65	Soft tissue inflammation
9	M	63	Cancer of unknown primary
10	M	62	Soft tissue inflammation
11	M	81	Staging completion
12	M	56	Staging completion
13	M	60	Staging completion
14	M	51	Staging completion
15	F	55	Staging completion
16	M	49	Staging completion
17	M	59	Soft tissue inflammation
18	M	50	Soft tissue inflammation
19	M	65	Staging completion
20	F	74	Staging completion
21	F	70	Staging completion
22	F	57	Staging completion
23	F	53	Staging completion
24	M	38	Cancer of unknown primary
25	M	43	Staging completion
26	M	56	Staging completion
27	F	57	Soft tissue inflammation
28	M	67	Soft tissue inflammation
29	F	73	Staging completion
30	F	30	Staging completion
31	M	77	Staging completion
32	M	72	Staging completion
33	M	79	Staging completion
34	F	66	Staging completion
35	F	58	Staging completion
36	M	38	Staging completion
37	F	66	Soft tissue inflammation
38	M	49	Soft tissue inflammation
39	M	64	Staging completion
40	F	68	Soft tissue inflammation
DECT group			
41	M	79	Staging completion
42	M	79	Soft tissue inflammation
43	M	73	Staging completion
44	M	62	Staging completion
45	F	80	Soft tissue inflammation
46	F	56	Staging completion
47	M	52	Staging completion
48	F	69	Soft tissue inflammation
49	F	66	Staging completion
50	M	38	Staging completion
51	M	56	Staging completion
52	M	77	Staging completion
53	M	52	Cancer of unknown primary
54	M	58	Staging completion
55	M	67	Staging completion
56	F	71	Staging completion
57	M	71	Staging completion
58	M	52	Staging completion
59	M	74	Soft tissue inflammation
60	M	61	Soft tissue inflammation
61	M	73	Soft tissue inflammation
62	F	67	Staging completion
63	F	78	Soft tissue inflammation
64	M	75	Soft tissue inflammation
65	M	74	Staging completion
66	F	68	Staging completion
67	M	78	Staging completion
68	F	52	Staging completion
69	F	73	Soft tissue inflammation
70	M	59	Oral cancer
71	M	48	Soft tissue inflammation
72	M	63	Staging completion
73	F	49	Soft tissue inflammation
74	F	49	Staging completion
75	M	55	Soft tissue inflammation
76	M	58	Staging completion
77	M	80	Staging completion
78	F	74	Soft tissue inflammation
79	F	81	Soft tissue inflammation
80	M	87	Staging completion

### CT technique and acquisition protocol

All examinations were performed on a third-generation dual-source CT scanner (Siemens SOMATOM Force, Siemens Healthcare GmbH) equipped with two 192-row energy integrating detectors (Siemens Stellar-Infinity, Siemens Healthcare GmbH) arranged in an orthogonal direction. The scanner provides a maximum of 240 kW generator power (2 × 120 kW).

In both study groups, CT acquisition of the neck was performed with both arms lowered and placed beside the trunk. After performing the localizer and determining the field of view, 80 mL of iodine contrast agent (Imeron 350 mg/mL, Bracco GmbH) was intravenously injected at a flow rate of 3 mL/s, followed by a saline bolus (30 mL, 3 mL/s). Both the DECT and the SECT examination protocols were performed after a fixed delay of 80 s for all study participants. A real-time automatic tube current modulation algorithm (CARE Dose4D, Siemens Healthcare GmbH) was used for all examinations in both study groups.

### Single-energy CT acquisition

An automatic TVA algorithm (CARE kV, Siemens Healthcare GmbH) was used for the SECT study group. TVA is capable of tube voltage settings from 70 to 150 kV in discrete steps of 10 kV. A preselection of TVA presets for different applications is available and can be directly adjusted by the user on a 12-point slider. In this study, a mid-level TVA setting (slider position 7 of 12) was chosen to keep the CNR balanced. To avoid excess radiation dose, TVA was limited from 70 to 120 kV.

### Dual-energy CT acquisition

DECT acquisition was performed with tube voltages of 80 kV (tube A) and 150 kV (tube B). Both x-ray beams were pre-filtered with an aluminum bowtie filter, with tube B equipped with an additional 0.5-mm tin prefiltration in order to refine the spectral separation. Table 2 provides a detailed overview of the acquisition and reconstruction parameters for the SECT and DECT groups.

**Table 2:** Scan parameters and image reconstruction settings for the dual-energy CT (DECT) and single-energy CT (SECT) groups

Scan parameters	DECT	SECT
Tube voltage (kV)	Tube A: 80 Tube B: 150 (tin prefiltration)	Range: 70–120
Reference mAs	Tube A: 91 Tube B: 61	200
Pitch	0.8	0.8
Reconstruction kernel (soft tissue)	Bf40	Bf40
Matrix	512 × 512	512 × 512
Collimation	192 × 0.6 mm	192 × 0.6 mm
Slice thickness	3 mm	3 mm
Increment	3 mm	3 mm
Iterative reconstruction	No	No
Rotation time	0.5 s	0.25 s
CTDI_vol_ (mGy; mean)	18.1	18.6
Average scan length	32.1 cm	32.4 cm
Exposure time	0.625 s	0.3125 s
Grayscale depth (bit)	12	12

### Weighted average images

Based on DECT raw data, WAI series were reconstructed in-line on the scanner console with a slice thickness of 3 mm in axial orientation and soft (Bf40) and sharp (Br64) convolution kernels. In principle, WAIs are intended to provide image quality equivalent to that of conventional SECT reconstructions. A multiband filtered algorithm was applied to DECT raw data as recommended by the vendor (F-type reconstruction 0.7, nonlinearly merging 70% of the 80 kV and 30% of the 150 kV data spectra).

### Single-energy CT images

Like the WAIs, SECT data were reconstructed with 3-mm slice thickness in axial orientation using equivalent soft (Bf40) and sharp (Br64) convolution kernels. Convolution kernels for WAI and SECT images were vendor matched to ensure comparability. Concordant to WAI, no iterative reconstruction algorithm was used in order to limit the bias between SECT and DECT images.

### Objective image quality

**Fig. 2:**
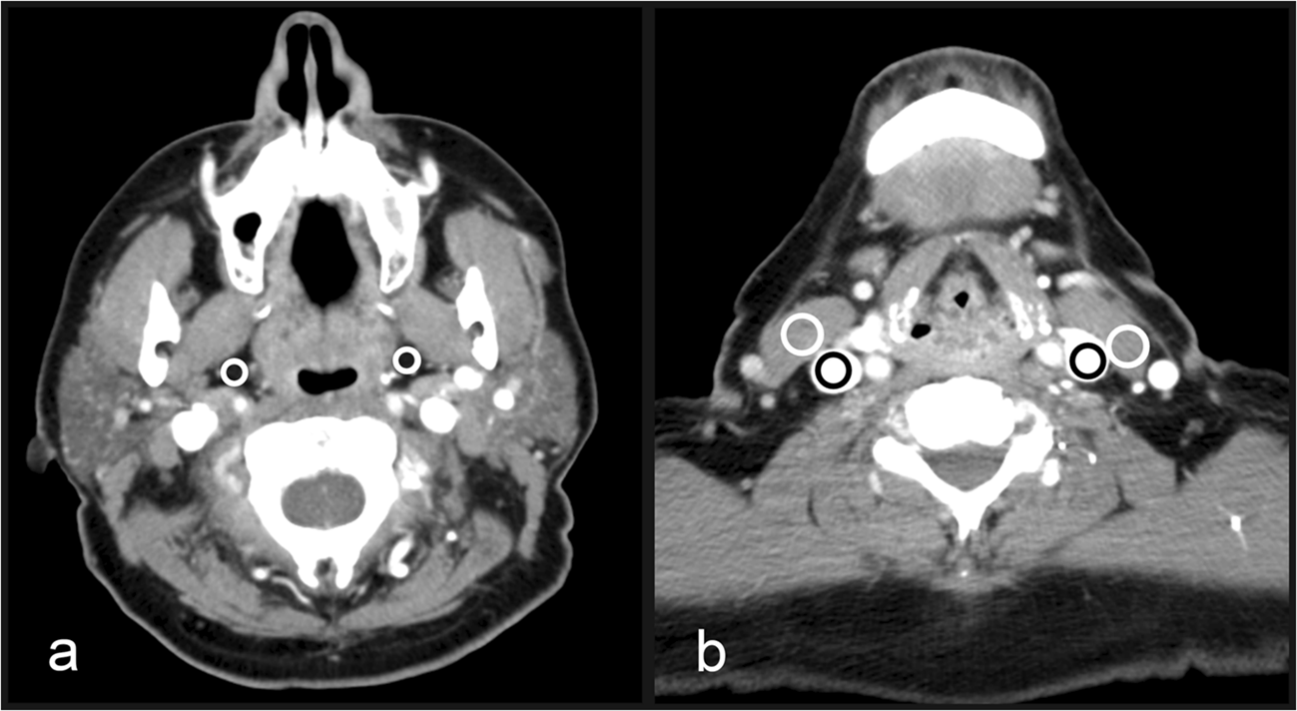
Image illustrating the regions of interest drawn in predefined anatomic structures. Image from a 68-year-old female patient who underwent single-energy CT. The image on the left side (**a**) shows the level chosen for measurement in the parapharyngeal space (marked as white circles), and the image on the right side (**b**) depicts the level chosen for measurement in the internal jugular veins (marked as black circles) and the adjacent sternocleidomastoid muscles (marked as white circles). Regions of interest were carefully drawn to avoid artifacts from dental hardware

After anonymization, evaluation of image quality was performed on a dedicated workstation with a server/client-based software (Syngo.via VB 20, Siemens Healthcare GmbH) by two board-certified radiologists with 6 and 11 years of experience in head and neck imaging. All imaging series were presented in a randomized order, and both readers were blinded to other imaging data and the patients’ medical history. Regions of interest (ROIs) were placed on axial slices in both jugular veins, in both sternocleidomastoid muscles, and in both fat-containing parapharyngeal spaces. All ROIs were manually drawn as large as possible while carefully avoiding the inclusion of neighboring structures and artifacts. Reproducibility was ensured by defining specific anatomic positions on the axial slices: ROIs in jugular vein and sternocleidomastoid muscle were drawn between the lower boundary of the mandible and the hyoid bone, ROIs in the parapharyngeal space were drawn between the angle and the head of the mandible. In both positions, the readers had to check for artifacts before ROI measurements were performed. See Fig. 2 for an illustration of ROI placement. In order to obtain objective parameters of image quality, mean attenuation (*A*) of every ROI was measured in Hounsfield units (HU). Fatty tissue within the parapharyngeal space was used as the reference for image noise (*N*) by measuring the standard deviation of the mean attenuation. Image review began with a default soft tissue (center 50 HU/width 400 HU) and bone window (450/1500 HU) that could be adjusted at the reader’s discretion. CNR was calculated using Eq. 1 and dose-normalized CNR (CNRD) was calculated using Eq. 2.

1 $$ \mathrm{CNR}=\left({A}_{\mathrm{value}\ 1}-{A}_{\mathrm{value}\ 2}\right)/{N}_{\mathrm{fat}} $$

2 $$ \mathrm{CNRD}=\mathrm{CNR}/\surd {\mathrm{CTDI}}_{\mathrm{vol}} $$

### Subjective image quality

Sixteen anatomical substructures were evaluated using a default soft tissue window setting (center 50 HU/width 400 HU) according to the European Guidelines on Quality Criteria for CT [10]. The substructures analyzed were as follows: pharynx (1: wall of the pharynx, 2: mucosal margins, 3: parapharyngeal fat spaces, 4: parapharyngeal muscles), larynx (5: wall of the larynx, 6: mucosal folds, 7: perimucosal fat spaces, 8: intrinsic laryngeal muscles, 9: paralaryngeal muscles), salivary glands (10: glandular tissue, 11: margins of normal glands, 12: paraglandular fat spaces, 13: mandible and associated muscles), and lymphatic tissue (14: regional lymph node areas of the pharynx, 15: regional lymph node areas of the larynx, 16: regional lymph node areas of the salivary glands). Overall image noise, spatial resolution, and diagnostic acceptability were also evaluated.

In compliance with the guidelines mentioned above, each reader assessed the visually sharp reproduction of anatomical substructures on a two-point Likert-type scale (1: no, 2: yes), the image noise and spatial resolution on a three-point Likert-type scale (1: too little, 2: optimum, 3: too much), and the overall diagnostic acceptability on a four-point Likert-type scale (1: fully confident for diagnostic interpretation, 2: probably confident for interpretation, 3: confident only under limited conditions for visualization of abnormalities, 4: unacceptable). Image artifacts were also graded on a four-point Likert-type scale (1: no artifacts, 2: minor artifacts not affecting the visualization of any structure, 3: major artifacts affecting the visualization of normal structures, 4: artifacts affecting diagnostic information). Beam hardening and photon starvation artifacts caused by metal dental hardware were not evaluated. Inter-rater agreement was calculated based on the results obtained for each category and each acquisition technique.

### Radiation exposure

The radiation exposure was quantified as pitch-corrected computed tomography dose index (CTDI_vol_) and dose length product (DLP) as provided by the scanner. The effective radiation dose (ED) associated with the CT examination was calculated using the specific conversion factor for neck examinations in adults (0.0051 mSv × mGy^−1^ × cm^−1^) [11]. The estimated radiation exposure in the SECT group was matched to that of the DECT group in an ex ante trial using a dedicated 16-cm acrylic CTDI phantom by adjusting the reference tube current time product stepwise. Single-energy examinations were referenced to a 32-cm phantom; thus, values had to be converted to match the 16-cm phantom [11]. According to the vendor (Siemens Healthcare GmbH), the respective conversion factor for the CT system used in this study is 2.0 for 120 kV, but it must be adapted according to the chosen kilovolt value by TVA to a maximum of 2.4 at 70 kV.

### Statistical analysis

Interval-level data were evaluated for normal distribution using the Shapiro–Wilk test. If the data were assumed to be normally distributed, values are given as mean ± standard deviation; otherwise, and in cases of ordinal-level data, values are given as median and interquartile range. For comparison of objective image quality data and radiation exposure between the SECT and DECT groups, a non-parametric Mann–Whitney *U* test was performed, as normal distribution could not be assumed according to the Shapiro–Wilk test. Comparison of subjective image quality ratings was performed using a Wilcoxon rank sum test. Inter-rater agreement was evaluated by calculating Cohen’s kappa value (*κ*); *κ* was interpreted according to Landis and Koch [12]. Results were accepted as statistically significant for *p* values < 0.05. The Statistical Package for the Social Sciences (SPSS) was used for randomization process and data analysis (IBM SPSS for Windows, Version 24.0 released 2016, IBM Corp.).

## Results

In the SECT group, the study population consisted of 16 female and 24 male patients with a mean age of 62 ± 12 years, while the DECT group consisted of 14 female and 26 male patients with a mean age of 66 ± 12 years.

### Objective image quality

In SECT and DECT, ROIs for attenuation analysis could be drawn in all study participants (Fig. 2). Mean ROI size was 0.47 ± 0.18 cm^2^ for vessel attenuation, 0.60 ± 0.13 cm^2^ for muscle attenuation, and 0.33 ± 0.15 cm^2^ for parapharyngeal fatty tissue. For DECT, a significantly increased CNR and CNRD was found for jugular veins relative to fatty tissue and for muscle tissue relative to fatty tissue compared to SECT (all *p* < 0.001). No significant difference was found for vessel attenuation relative to muscle tissue (CNR *p* = 0.36; CNRD *p* = 0.35). Table 3 summarizes all results from the objective image quality assessment.

**Table 3:** Detailed overview of objective image quality data for the single-energy CT and dual-energy CT groups. Statistically significant *p* values are marked with an asterisk

Group	SECT	DECT	*p* value
	Attenuation values; mean ± standard deviation		
Jugular vein	285 ± 101	229 ± 54	
Muscle	88 ± 11	82 ± 9	
Fat	− 92 ± 21	− 106 ± 17	
	Image noise; mean ± standard deviation		
Fat	14 ± 3	11 ± 3	
	CNR median (interquartile range)		
Jugular vein–fat	25.0 (10.1)	30.1 (9.6)	< 0.001*
Jugular vein–muscle	11.9 (8.7)	12.6 (5.5)	0.36
Muscle–fat	12.2 (5.4)	17.1 (5.6)	< 0.001*
	CNRD median (interquartile range)		
Jugular vein–fat	5.9 (2.6)	6.9 (2.7)	< 0.001*
Jugular vein–muscle	2.8 (2.1)	3.0 (1.5)	0.35
Muscle–fat	2.7 (1.1)	3.9 (1.7)	< 0.001*

### Subjective image quality

All anatomical substructures evaluated were visually sharp and definable in all SECT and DECT examinations.

**Fig. 3:**
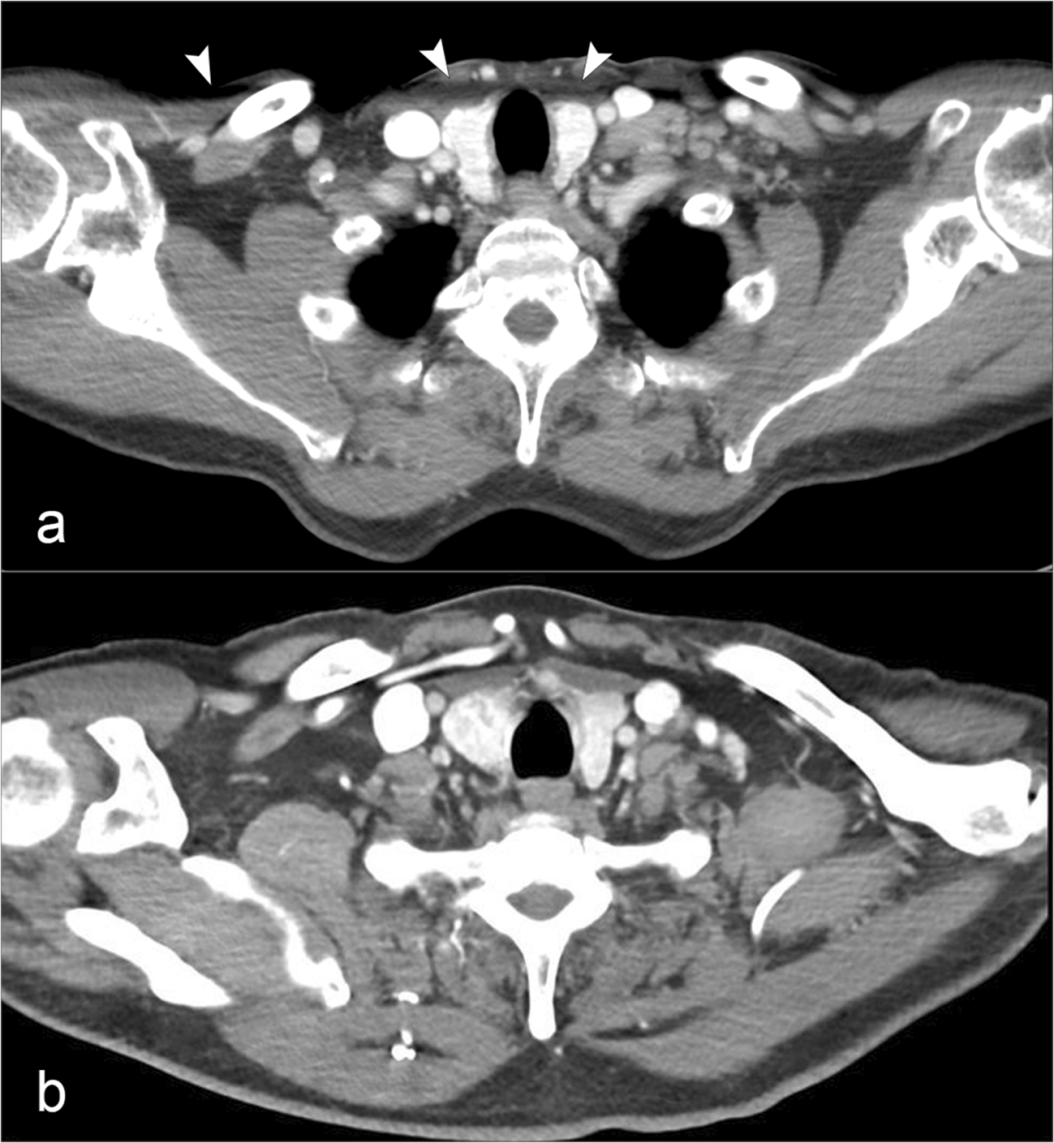
Streak artifacts at the cervicothoracic transition zone in single-energy CT (SECT). Streak artifact (white arrows) found at the level of the shoulders with the SECT technique. Images are from a 63-year-old male patient with a cT2N0 carcinoma of the oropharynx who underwent SECT at 70 kV (**a**) and a 52-year-old male patient who underwent dual-energy CT (DECT) for staging completion (**b**). All images are shown in the same window setting (center = 50 HU, width = 500 HU). No streak artifacts were found with DECT at the height of the shoulders

Image noise and spatial resolution were comparable between DECT and SECT for both readers. In contrast, overall diagnostic acceptability and image artifacts differed significantly between the DECT and SECT groups in favor of DECT for both readers. See Table 4 for a detailed overview and specific *p* values. Figure 3 provides a sample comparison of artifacts at the height of the shoulders between SECT and DECT.

**Table 4:** Total sum of Likert scores (image noise, overall diagnostic acceptability and image artifacts: lower sum indicates better performance; spatial resolution: higher sum indicates better performance) and calculated *p* values for each rating category per reader. Significant *p* values are marked with an asterisk

	Reader 1			Reader 2		
	DECT	SECT		DECT	SECT	
Image noise	83	91	*p* = 0.7	81	89	*p* = 0.8
Spatial resolution	82	83	*p* = 0.7	82	84	*p* = 0.5
Overall diagnostic acceptability	70	90	*p* = 0.01*	72	91	*p* = 0.01*
Image artifacts	94	118	*p* = 0.01*	92	114	*p* = 0.01*

Inter-rater agreement for image noise evaluation, overall diagnostic acceptability, and image artifacts was substantial for SECT and DECT (*κ* > 0.7). A moderate inter-rater agreement was found for spatial resolution (SECT and DECT *κ* = 0.6).

### Radiation exposure

In the SECT group, TVA automatically selected 70 kV in 3 of the 40 patients (7.5%), 80 kV in 10 patients (25%), 90 kV in 19 patients (47.5%), and 100 kV in 8 patients (20%). Neither 110 nor 120 kV was selected. CTDI_vol_, DLP, and ED were comparable between the study groups. Table 5 provides a detailed overview.

**Table 5:** Detailed overview of parameters characterizing radiation exposure of the single-energy CT and dual-energy CT study groups

	SECT	DECT	*p* value
CTDI_vol_	18.4 ± 2.9 mGy	18.5 ± 4.1 mGy	0.7
DLP	582 ± 78 mGy*cm	603 ± 103 mGy*cm	0.5
ED	2.9 ± 0.6 mSv	3.1 ± 0.7 mSv	0.5

## Discussion

In our study, WAI delivered superior CNR and CNRD values for the sternocleidomastoid muscle relative to fat and for the jugular vein relative to fat in head and neck imaging compared to dose-matched standard SECT images with automated TVA. Image noise and spatial resolution were comparable between the study groups, but WAIs had fewer image artifacts and better overall diagnostic acceptability than SECT images.

Head and neck imaging is especially challenging due to the relevant anatomic structures being in close proximity and low intrinsic contrast. For lesion detection and exact anatomic localization, image contrast must be maximized while image noise is minimized. To achieve a homogeneous distribution of noise over the scan range while complying with the “as low as reasonably achievable” (ALARA) principle in radiation protection, TCM is commonly implemented in all modern CT scanners. The tube current is automatically decreased in projections of low attenuation and increased in projections of high attenuation (like the cervicothoracic transition).

DECT acquires one data set, but allows for various post-processing options to simulate different energy levels and thereby generate different CNRs. Many studies have investigated the effect of using low-kilo-electron-volt VMIs like 40 keV to increase CNR, but direct comparison between low-kilovolt SECT and DECT is rare in head and neck imaging [7, 13, 14]. In a recently published study, SECT images with a fixed 70-kV setting were comparable to 40-keV VMI reconstructions for tumor delineation; however, the study focused on a limited scan region, excluding critical regions like the cervicothoracic transition [15]. Thus, in SECT, low-kilovolt scanning like 70 kV is suitable for soft tissue evaluation covering only parts of the neck, but its use is limited for challenging regions like the cervicothoracic region or the oral cavity, which may contain artifacts due to dental implants [16].

This is also reflected in the automatically selected tube voltages in our study. Only three patients were scanned with 70 kV; higher kilovolt values were used in over 90% of the patients. These settings, based on the patient anatomy, seem to be the best compromise between higher iodine attenuation and higher image noise in SECT. DECT delivered superior soft tissue contrast, as reflected by the CNR and CNRD values for sternocleidomastoid muscle relative to fat and for the jugular vein relative to fat. This is due to WAIs representing a blend of high- and low-energy information and thereby combining higher image contrast from low-kilovolt levels with lower image noise levels from higher-kilovolt levels (in our study, nonlinearly merging 70% of the 80-kV and 30% of the 150-kV data spectra). DECT not only improved objective parameters like CNRD, but also exhibited higher subjective image quality compared to SECT.

Future developments, such as instantly available low-kilo-electron-volt levels with no additional post-processing, will further establish the use of DECT, especially in anatomical regions like the head and neck that suffer from low intrinsic contrast.

## Limitations

Some limitations must be considered when interpreting the results of our study:

- First, due to ethical reasons, we could not perform an intra-individual comparison between SECT and DECT.
- Second, only one vendor’s dual- and single-energy options were evaluated, and no direct comparison to other CT scanners is possible.
- Third, dedicated follow-up trials must be performed to investigate whether the higher image quality of WAIs compared to SECT images also translates into increased diagnostic accuracy of lesion detection.
- Fourth, no iterative reconstruction algorithms were used and evaluated in this study. Further studies may show additional benefits of iterative reconstruction algorithms.

## Conclusion

DECT with instantly availably WAI delivers superior image quality compared to SECT with tube voltage adaptation and is beneficial for head and neck imaging, which suffers from low intrinsic contrast.

## Abbreviations

CNR: Contrast-to-noise ratio
CNRD: Dose-normalized contrast-to-noise ratio
CT: Computed tomography
HU: Hounsfield units
ROI: Region of interest
SNR: Signal-to-noise ratio
STD: Standard deviation
TCM: Tube current modulation
TVA: Tube voltage adaptation

## Declarations

### Guarantor

The scientific guarantor of this publication is Dr. Marco Wiesmueller.

### Statistics and biometry

No complex statistical methods were necessary for this paper.

### Methodology

• prospective

• diagnostic study

• performed at one institution
